# Time optimization in primary care – chronic prescription cost

**DOI:** 10.1186/s12913-023-09355-1

**Published:** 2023-05-08

**Authors:** Tiffany Leite-Costa, Daniel Rodrigues, Fernando Sá, Ricardo Cruz Correia

**Affiliations:** 1 Mestrado em Informática Médica, Universidade do Porto, Porto, Portugal; 2 USF Covelo, ACeS Porto Oriental, Porto, Portugal

**Keywords:** Eletronic prescription, E-prescribing system, Time optimization, Primary care, Health costs

## Abstract

**Introduction:**

Time optimization is a common goal to most health information institutions. In several countries, chronic electronic renewal prescriptions were one of the main focuses when implementing information systems. In Portugal, Electronic Medical Prescription (PEM®) software is used for most electronic prescriptions. This study aims to quantify the time spent in chronic prescription renewal appointments (CPRA) in primary care and its impact in the Portuguese National Health System (SNS).

**Methods:**

Eight general practitioners (GP) were included in the study during February 2022. The average duration of 100 CPRA was obtained. To determine the number of CPRA performed every year, a primary care BI-CSP® platform was used. Using Standard Cost Model and average medical doctor hourly rate in Portugal we estimated CPRA global costs.

**Results:**

Each doctor spent on average 1:55 ± 01:07 min per CPRA. There were 8295 GP working in 2022. A total 635 561 CPRA were performed in 2020 and 774 346 in 2021. In 2020, CPRA costs ranged 303 088 ± 179 419€, and in 2021 that number increased to 369 272 ± 218 599€.

**Conclusion:**

This is the first study to quantify CPRA’s real cost in Portugal. A PEM® software update would allow daily savings, ranging from 830€ (± 491€) in 2020 and 1011€ (± 598€) in 2021. That change could allow hiring 8 ± 5 GP in 2020 and 12 ± 7 in 2021.

## Introduction

Optimizing time in primary care is one of the Organization for Economic Co-operation and Development´s (OECD) main concern [1]. Managing health workers time accurately influences waiting periods for medical observations, especially in an environment where health professionals are lacking. Electronic medical prescribing software developers aim to increase security while diminishing time spent on this task. However, when using them, its users pose some constraints regarding the number of steps required to complete an assignment [2].

There are multiple electronic prescribing platforms (EPP) available worldwide depending on the country and degree of development. The European Union aims to achieve uniformity and interoperability between EPP by 2025 [3]. Their main advantages include patient’s sense of security, development of clinical decision support systems and pharmacologic interactions or allergic reactions safety alerts implementation [4]. Other possible positive aspects include better vigilance on patient treatment adherence [5, 6] and workflow efficiency [7]. A study showed a 50% waiting time reduction for chronic medication prescriptions following EPP implementation in the United States [7].

Nevertheless, there are systematic concerns that linger, even after the initial learning and transition phases. A team of American investigators evaluated a group of clinicians satisfied with the EPP used and verified that only 2 years after implementing it, the clinicians were as effective as before [8]. A Finnish workgroup found that associated bureaucracy when using EPP was a main concern for professionals that consider chronic prescription renewal a non-medical act occupying a significant amount of time [2].

There are no published studies evaluating time dedicated to chronic e-prescription renewal appointments (CPRA) or its economic cost, as there are none regarding Portuguese electronic prescription in reality. Since 2013, Electronic Medical Prescription (PEM®) software is used in most Portuguese health institutions [9].

In Portugal, chronic e-prescribing appointments are non-face-to-face consultations that patients request either by phone, email or in some cases in person, to renew chronic prescriptions they lack until the next face-to-face appointment. They do not involve a personal contact with the physician. Patients can either come back to collect the paper e-prescription version or use a text message code in the pharmacy directly. Usually, these chronic e-prescriptions are valid for 6 months and are used for chronic medication for hypertension, diabetes, heart, pulmonary and/or kidney diseases.

The Standard Cost Model (SCM) is an OECD recommended method to determine administrative workload generated by an activity. It helps to quantify administrative impositions’ global impact on a task [10]. SCM separates an assignment in different parts translating it to costs through the formula: Hourly rate x Time x Quantity [11].

The aim of this study is to evaluate the average time spent by general practitioners (GP) in CPRA. We intend to estimate the possible economic benefits of optimizing e-prescribing procedures.

## Methods

Data regarding CPRA duration in a family health unit was collected. To obtain the best reliability and patient-doctor confidentiality, resident doctors recorded CPRA length. To assure measurement homogeneity, all resident doctors were instructed with demonstrations of appointment start and finish. These timings and recordings were evaluated using the same chronometer mobile app. All CPRA were based on the PEM® software.

After data collection, a CPRA average interval was calculated and transformed into costs using SCM model according to the formula:

CPRA Number x GP average hourly rate (euros) x CPRA average time (hours).

To determine total CPRA numbers, the national primary care database (BI-CSP) was used. CPRA codes and its variants were selected. These numbers were registered for two consecutive years: 2020 and 2021 [12].

To determine average GP hourly rate, we collected national data through the BI-CSP database regarding the physicians professional degrees and primary health centers, by the end of February 2022. The last published salary tables were used to determine average GP hourly rate given the physicians professional degree and type of health center [12].

The average costs each year and savings per day with PEM® update were calculated and converted into human resources hired per year.

## Results

The duration of 100 different CPRA were obtained. Eight different doctors were timed: three 1st degree residents, one 2nd degree resident, two medical assistants, one graduated medical assistant and 1 senior graduated medical assistant. Per CPRA, each professional spent on average 1 min and 55 s (± 01:07.998) or the equivalent to 0.032 h (± 0.019) (Table 1).

**Table 1:** Cost and time spent in CPRA in 2020 and 2021

Parameter	2020	2021
*CPRA Number – n (%)*:	635 561 (± 1,3)	774 346 (± 1,2)
*Estimated time spent in CPRA – hours/year (DP)*:	20 308 (± 12 022)	24 743 (± 14 647)
*Estimated CPRA cost per year – €/year (DP)*:	303 088 (± 179 419)	369 272 (± 218 599)
Estimated savings with PEM update – *€/day (DP)*:	830 (± 491)	1011 (± 598)
Estimated number of GP hired with PEM update – GP/year (DP):	8 (± 5)	12 (± 7)
Estimated time spent per CPRA:		
*min:sec.ms (DP)*	01:55.220 (01:07.998)	
*Hours (DP)*	0,032 (0,019)	

In February 2022 there were 8295 physicians working in primary care. Of these, 27.8% were residents, 27.5% worked in model B health centers, 21% in model A and 23.7% worked in personalized health center units (PHCU). Medical professional degree distribution and average hourly rate are presented in Table 2.

**Table 2:** Medical doctors’ distribution through professional degree nationwide and hourly rate

Variables	n (%)	Variables	n (%)
Model B general practitioners:		PHCU general practitioners:	
*Graduated Assistant*	1380 (45,6)	*Graduated Assistant*	608 (30,9)
*Graduated Senior Assistant*	82 (3,6)	*Graduated Senior Assistant*	53 (2,7)
*Medical Assistant*	1119 (49,1)	*Medical Assistant*	830 (42,2)
*Others*	39 (1,7)	*Others*	478 (24,3)
*Total*	2278 (27,5)	*Total*	1969 (23,7)
Model A general practitioners:		Primary Care Residents:	
*Graduated Assistant*	436 (25,1)	*1st Degree*	1440 (62,3)
*Graduated Senior Assistant*	44 (2,5)	*2nd Degree*	870 (37,7)
*Medical Assistant*	1148 (66,1)	*Total*	2310 (27,8)
*Others*	110 (6,3)		
*Total*	1738 (21)		
*Hourly rate - €/h*:			
*Model B GP*		22,53	
*Model A GP*		12,10	
*PHCU GP*		12,88	
*1st Degree Resident*		10,62	
*2nd Degree Resident*		11,21	
*Portuguese GP Average*		14,92	

A total of 635 561 CPRA were performed in 2020 and 774 346 in 2021 (Table 1). After applying the SCM formula, estimated expenses of 303 088€ (± 179 419€) for 2020 and 369 272€ (± 18 599€) for 2021 were obtained. These appointments costed 830€/day (± 491€) in 2020 and 1011€/day (± 598€) in 2021. An estimate of 8 GP (± 5) in 2020 and 12 GP (± 7) in 2021 could have been hired given the established cost (Table 1).

## Discussion

In conclusion, it is believed that since its development and use, digital health has evolved rapidly, with large investments in health technologies [13]. However, frustration among stakeholders and health professionals arose given the lack of tangible benefits and an increase in administrative burden. Digital health transition should focus on humanizing technology and its experience for every intervenient [13].

According to the OECD, in 2017 health institution’s main goal was time mismanagement reduction and better use of resources when applying structural reforms in Health Systems, granting quality in healthcare [14]. It is estimated that 20% of health institution’s spendings are misused in the US alone [15].

Several articles have highlighted positive aspects of electronic prescribing transitioning from paper versions [16–20]. One of its benefits is improved accuracy and patient safety either by automatically checking for drug interactions, allergies, and other potential problems, or by providing alerts to doctors and pharmacists if there are any potential issues with a prescription, such as drug overuse.

E-prescribing can also enhance communication between healthcare providers, allowing doctors to easily share information about a patient’s medications with other providers, that can help improve the coordination of care [16, 17].

Another reported benefit is increased efficiency and reduced costs. Electronic prescribing systems allow doctors to write and transmit prescriptions electronically, which can save time and improve the efficiency of the prescribing process [19, 20]. In addition, e-prescribing can help reduce costs by reducing the need for paper prescriptions and by helping to prevent medication errors, which can be costly to treat [16–18]. Nonetheless, these findings are not absolute and most systematic reviews found lack of consensus and required further research [21–23].

In Portugal, the use of PEM® e-prescribing software is prevalent across the National Health Service, primary care centers and pharmacies. It represents an important evolution from its paper version with several advantages such as uniformizing medical prescription across sectors.

So far this is the only study presenting a national quantitative projection of the time dedicated to chronic e-prescription in Portugal. Other qualitative studies have evaluated the influence of EPP in other fields such as chronic respiratory care (PEM-CRD®) and established improvements in efficiency comparing to paper versions [24].

It is important to understand the perspectives of health workers regarding PEM®. Given the generalized bureaucracy dissatisfaction in healthcare settings, it is essential that technicians and health professionals work together to develop and implement digital solutions in a multidisciplinary context [25].

As previously mentioned, in Portugal, chronic e-prescribing usually does not implicate a personal contact between patient and physician. Currently it involves a paper version or a text message code which patients then take to a pharmacy. Several problems present with this system: patients often lose papers or delete text messages between appointments (specially with 6 months valid prescriptions). When this occurs, patients request a chronic prescription renewal appointment, and the physician repeats the process. Even though these appointments represent only 1.2–1.3% of primary care activities, each patient uses them on average 2.4 times beyond their personal encounter with the doctor [12].

This study established chronic prescription appointments duration with some variability. The economic return of an automatized solution was estimated in terms of cost and human resources. It would be possible to upgrade PEM® to deal with chronic prescriptions automatically without any additional investment or added encounters, saving time, cost, and software development. In its current form, doctors can “e-prescribe” during a routine appointment, but to fill in the prescription, the pharmacy requires either a paper version or a text message code even though they use the same PEM® platform.

Interoperability is considered a cornerstone in electronic prescription systems [26] allowing better communication between doctors and pharmacists. In its current state, PEM® is not a fully functional e-prescribing system and an upgrade is required to circumvent the need for a paper or a text message code. This alternative would not require further software development and would greatly impact primary care workflow. Besides the costs, there is a potential increase in professional satisfaction and medical error reduction [11]. However, possible unintended consequences must be considered, as there are several issues that a potential PEM upgrade should address: the potential for medication overuse, the way in which private practices or hospitals would be included, data security and privacy concerns regarding transmission and storage of sensitive patient information, and the integration between the public national health service and privately owned pharmacies. There is also the risk for unintended renewal.

There are some important limitations to this study. The number of CPRA refers to 2020 and 2021 data while the lockdown caused by Covid-19 was in course, and consequently these numbers could be overestimated. These values may not be applicable to the pre or post pandemic period. Additionally, costs were estimated with 2022 human resources data which were the only ones available. Income among different professional degrees was assessed with an average of the lowest and highest degrees, which might overestimate the average hourly rate due to the lack of career progression in Portugal [27]. Our sample included 100 appointments spread over 8 physicians, and even though the results are probably replicable in other health centers, it would be advisable to repeat this study with larger samples to have a better representation of national data. We did not compare e-prescription timing with previous methods (as traditional paper handwritten prescription), but previous trials have demonstrated that, over time, e-prescription allows a better overall performance [7].

## Conclusion

This is the first study to quantify the impact of CPRA nationwide and further studies, with a broader range of health centers involved are required to draw more reliable conclusions. Most importantly, both the problem and solution appear to be well addressed and feasible.

General practitioners spent an average of 1:55 ± 01:07 min per CPRA, which according to our model translates into an expense of 830€/day (± 491€) in 2020 and 1011€/day (± 598€) in 2021. This value could be saved with the upgrade suggested in this article, transforming an existing solution while reducing costs and increasing health workers’ and patients’ satisfaction.

## Data Availability

The data that support the findings of this study are available from *Bilhete de Identidade dos Cuidados de Saúde Primários* database, but it is required to be a government professional to have this data available, and so are not publicly available. Data are however available from the authors upon reasonable request and with permission of *Bilhete de Identidade dos Cuidados de Saúde Primários from Serviços Partilhados do Ministério da Saúde*, a publicly available version is found under the URL: https://bicsp.min-saude.pt/pt/Paginas/default.aspx.

## Declarations

### Duplicate policy

The content of the manuscript has not been published or submitted for publication elsewhere.
